# Determination of lamotrigine in human plasma by HPLC-PDA. Application to forensic samples

**DOI:** 10.1007/s12024-024-00812-9

**Published:** 2024-04-10

**Authors:** Inés Sánchez-Sellero, Iván Álvarez-Freire, Pamela Cabarcos-Fernández, Lidia Janza-Candal, María Jesús Tabernero-Duque, Ana María Bermejo-Barrera

**Affiliations:** Forensic Toxicology Service, Forensic Sciences Institute, Faculty of Medicine, Universidade de Santiago de Compostela, C/ San Francisco s/n, Santiago de Compostela, 15782 Spain

**Keywords:** Lamotrigine, Plasma, HPLC–PDA, Forensic

## Abstract

**Purpose:**

Therapeutic drug monitoring of plasma lamotrigine (LTG) has customarily been carried out in order to prevent some its adverse effects. For forensic purposes, determination of LTG in plasma is an useful tool in cases of accidental overdose or suicidal attempts. Currently, there are several analytical methods available including some based on LC tandem mass spectrometry techniques, but simple and accessible LC-UV methods still can be useful for the purpose. Here we report on a new high-performance liquid chromatography method for the determination of lamotrigine in human plasma which has been developed and validated including selectivity, sensitivity, accuracy, precision and recovery studies.

**Methods:**

Lamotrigine and the internal standard chloramphenicol were extracted from plasma using liquid-liquid extraction using small volumes of buffer and ethylacetate. Detection was monitored at 305.7 and 276.0 nm for lamotrigine and chloramphenicol, respectively.

**Results:**

The method was linear concentration dependence within the range of 0.1–10 µg/ml, with a mean coefficient of correlation *r* = 0.993. The limit of detection (LOD) was 0.04 µg/ml and the limit of quantification (LOQ) was 0.1 µg/ml. Intra and interday precision values were lower than 9.0% at all concentrations studied. The intra and interday accuracy values ranged from − 7.6 to 10.1%. Recovery was found to be 98.9% or higher. The method here described was successfully applied to 11 postmortem blood samples received at the Forensic Sciences Institute of Santiago de Compostela (Spain).

**Conclusion:**

A new HPLC method for the determination of lamotrigine in human plasma was developed and validated. A liquid-liquid extraction using small volumes of buffer and ethylacetate was optimized. The proposed method is suitable for forensic toxicological analysis.

## Introduction

Lamotrigine (LTG) [3,5-diamino-6-(2,3-dichlorophenyl)-1,2,4 triazine] is an antiepileptic drug of broad spectrum used in the adjunctive and monotherapy treatment of partial and generalized seizures as well as in the treatment of Lennox-Gastaut syndrome. Epilepsy is a common neurological disorder with a worldwide prevalence of around 1%. Lamotrigine is also used for treatment of bipolar disorder. Although lamotrigine was initially developed as an anti-folate drug, nowadays it has become one of the first-line antiepileptic drugs [1, 2]. Lamotrigine is a sodium ion channels modulator, enhancing their fast inactivation. The drug selectively binds and inhibits voltage-gated sodium channels, stabilizing presynaptic neuronal membranes and preventing the presynaptic release of glutamate and aspartate [3]. As a result of this action it blocks the action potential prolongation, stabilizes neuronal membranes and decreases the release of neurotransmitter, focal firing and seizure spread [4].

The inter- and intraindividual pharmacokinetic variability of LTG is a well-known fact and therefore individual dosing needs to be adjusted for each patient. The metabolism and elimination half-time of LTG can be influenced by some concomitant therapies with other medications. The LTG pharmacokinetics and metabolism could also be affected by several other conditions (i.e. pregnancy, diseases, age, genetic polymorphisms, renal replacement therapy) [5–8]. Several adverse effects of LTG, including rash and other life-threatening cutaneous reactions, have been reported. The fatality rate of severe cutaneous adverse reactions ranges from 10 to 35%. In addition, these reactions have the potential to cause disabilities [9]. Other adverse effects are hemophagocytic lymphohistiocytosis and blood dyscrasias. Nausea, vomiting, abdominal pain, ataxia, headache or visual disturbances are also been reported as side effects [3]. The LTG plasma concentrations may be increased by factors affecting its pharmacokinetics. High plasma levels of LTG could be related to an increased risk of adverse effects. Therapeutic drug monitoring of plasma LTG has been applied to minimize this risk. For forensic purposes, determination of LTG in plasma is also useful in cases of accidental overdose or suicidal attempts.

Lamotrigine is well absorbed via oral administration, and is approximately 55% bound to plasma protein. It undergoes glucuronidation and renal excretion, following first-order linear kinetics with a half-life of 24–30 h [3, 10]. The therapeutic plasma concentration of LTG was estimated to be in the range of 1–15 µg/ml and the toxic one in the range of 16–47 µg/ml. Although lamotrigine is extensively metabolized to glucuronide conjugates, most authors propose the determination of the parent drug rather than monitoring the glucuronide metabolite (2-*N*-glucuronide) [11]. Several methods for determination of LTG in biological fluids have been reported including high-performance liquid chromatography [6, 7, 10–19], liquid chromatography tandem mass spectrometry [5, 8, 20–24], and gas chromatography-mass spectrometry [25].

The aim of this study was to develop and validate a rapid, simple, sensitive, and specific high-performance liquid chromatography method for the quantitative determination of lamotrigine in plasma samples. Different extraction methods were tested and compared, and a liquid-liquid extraction procedure was finally selected. The analytical method was validated according to the guidelines of the Food and Drug Administration (FDA) [26]. The LOQ of this validated method was set at 0.1 µg/ml and the LOD at 0.04 µg/ml. The method proved to be suitable for the analysis of postmortem blood samples, when applied to samples received at the Forensic Toxicology Service of the Forensic Sciences Institute of Santiago de Compostela (Spain).

## Materials and methods

### Chemicals and reagents

Lamotrigine Cerilliant^®^ (1.0 mg/ml in 2 ml methanol) and the internal standard (IS) chloramphenicol (purity ≥ 98%) were supplied by Sigma-Aldrich (Texas, USA). Methanol and ethylacetate were HPLC-grade (Sigma-Aldrich, Missouri, USA). Acetonitrile was LC-MS-grade (Millipore, Darmstadt, Germany). Sodium carbonate anhydrous (Na_2_CO_3_) and sodium hydrogen carbonate (NaHCO_3_) were analytical grade and obtained from Panreac (Barcelona, Spain) and Merck (Darmstadt, Germany), respectively. Di-potassium hydrogen orthophosphate trihydrate (K_2_HPO_4_.3H_2_O, purity ≥ 99%) and potassium dihydrogen orthophosphate (KH_2_PO_4_, purity ≥ 99%) were analytical grade and purchased from Merck (Darmstadt, Germany). Ultrapure water was obtained from a MilliQ-A10 apparatus (Millipore, Bedford, MA, USA).

### Blood samples

For validation purposes, drug-free human plasma samples were obtained from the Blood Bank of Santiago de Compostela (Spain). The developed method was applied to the analysis of plasma samples obtained from postmortem cases. Such samples were stored at − 20 °C until use. Plasma was obtained from whole blood by centrifugation (14,000 rpm for 5 min) and kept at 4 °C.

### Standard solutions

Stock solutions of LTG of 100, 10 and 0.1 µg/ml were prepared by dissolving lamotrigine 1.0 mg/ml in HPLC grade methanol. The stock solution of IS chloramphenicol (100 µg/ml) was prepared by dissolving the pure compound in HPLC grade methanol. All standard solutions were stored at -20 °C until use. Stock solutions of LTG were used to spike aliquots of blank human plasma to obtain the calibration standard solutions at concentrations of 0.1, 0.2, 0.5, 1, 2, 5 and 10 µg/ml LTG. Eight independent calibration samples were prepared and analyzed on separate days. Quality control (QC) samples of LTG at three concentration levels, namely low level QC (QC_1_ = 0.1 µg/ml), medium level QC (QC_2_ = 1 µg/ml), and high level QC (QC_3_ = 10 µg/ml), were also freshly and independently prepared in five replicates in the same biological matrix.

### Instrumentation and chromatographic conditions

The chromatographic analysis was carried out using an HPLC system (Waters 2695 Separations Module) coupled with a PDA (Waters^™^ 996 Photodiode Array Detector) (Waters, Milford, MA, USA). Instrumental parts were controlled and data processed by Empower^™^ 2 software (Build 2154 Waters). The chromatographic separation was carried out at room temperature on a reversed-phase XBridge^®^ Shield RP18 column (4.6 × 250 mm i.d., 5 μm particle size).

An isocratic elution was applied at a flow rate of 1 ml/min with a mobile phase consisting of acetonitrile-phosphate buffer (pH 6.5; 1mM) (30:70, v/v). Before use, the mobile phase was filtered through a 0.22 μm filter and degassed ultrasonically by using a Millipore (Waters) vacuum system. The injection volume was 20 µl and run time was 10 min. Detection was monitored at 305.7 and 276.0 nm for LTG and chloramphenicol, respectively.

### Sample preparation and extraction

The sample extraction procedure was optimized after a comparative study of different extraction methods, namely solid-phase extraction (SPE), ion liquid-based dispersive liquid-liquid microextraction (IL-DLLME), and liquid-liquid extraction (LLE). The final conditions of the optimized procedure of LL extraction were as follows. To 250 µl aliquots of plasma samples were added 25 µl of IS (chloramphenicol), 250 µl of Na_2_CO_3_-NaHCO_3_ buffer (pH 10) and 2 ml of ethylacetate. The samples were vortexed for 10 min and centrifuged at 3500 rpm for 5 min. The aqueous phase was discarded, and the organic layer was recovered and transferred to a conical tube and evaporated to dryness at 40ºC under a gentle flow of nitrogen. The residue was then reconstituted with 100 µl mobile phase, vortexed and transferred to an autosampler vial for HPLC-PDA analysis.

### Method validation

The analytical method developed was validated according to the FDA guidelines [26]. This method was validated regarding selectivity, linearity, limit of detection (LOD) and quantification (LOQ), intra and interday precision, accuracy, and recovery.

Selectivity of the developed method was assessed by analyzing ten blank plasma samples from different untreated individuals in order to evaluate the possible existence of interference from endogenous matrix substances.

Linearity was assessed by constructing eight calibration curves, processed on different days. Each calibration curve was performed after preparing and analyzing standard solutions at seven different concentration levels, ranging from 0.1 µg/ml to 10 µg/ml, including the LOQ. Calibration data were plotted as a graph of peak area ratios of LTG to internal standard as a function of the LTG concentration. Data were subjected to linear regression analysis, and calibration curves equations were calculated by least-squares method.

The LOD (lower limit of detection) was calculated at a signal-to-noise ratio of 3 (S/*N* = 3). The LOQ (limit of quantitation), defined as the lowest concentration of the calibration curve that can be measured with acceptable intra and interday precision and accuracy, was calculated at a signal-to-noise ratio of 10 (S/*N* = 10).

Precision of the proposed method was established by determining the relative standard deviation (%RSD = 100 × SD/average). Blank samples were spiked with LTG to obtain three quality controls (QCs) at low level (QC_1_ = 0.1 µg/ml), medium level (QC_2_ = 1 µg/ml), and high level (QC_3_ = 10 µg/ml), within the linear range of the calibration curve of lamotrigine. Interday precision was determined based on the analysis of three freshly prepared QC samples on five different days, whereas intraday precision was assessed by processing five replicates of each QC sample on the same day. Accuracy was calculated through relative error (%*bias*), following the same schedule as precision. According to the acceptance criteria, error values of accuracy and precision should not exceed 15% for each calibration standards, except at LOQ, where 20% of error is accepted.

Recovery experiments were performed with extracted QC samples at the three mentioned concentration levels in five replicates, by comparing the analytical results of these extracted plasma samples with corresponding extracts of blanks spiked with LTG post-extraction, following FDA recommendations [26].

## Results

### Selection of sample extraction procedure

Three extraction procedures, solid-phase extraction (SPE) [27], ion liquid-based dispersive liquid-liquid microextraction (IL-DLLME) [15], and liquid-liquid extraction (LLE) were tested and compared. In all of them, plasma samples spiked with the same amount of LTG (50 µl, 5 µg/ml) and IS (10 µl, 100 µg/ml) were used. The final sample extraction conditions were those previously mentioned in *materials and methods* section.

### Choice of internal standard

Chloramphenicol was successfully tested and used as internal standard in our method.

### Chromatographic separation

**Fig. 1:**
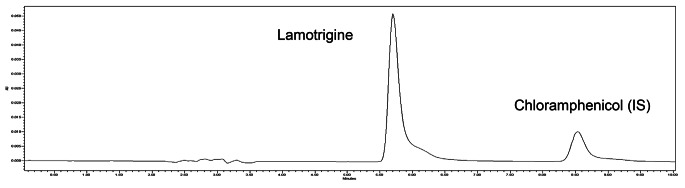
Chromatogram of a standard solution of lamotrigine (LTG) and chloramphenicol (IS). Retention times of 5.7 and 8.5 min for LTG and IS, respectively

**Fig. 2:**
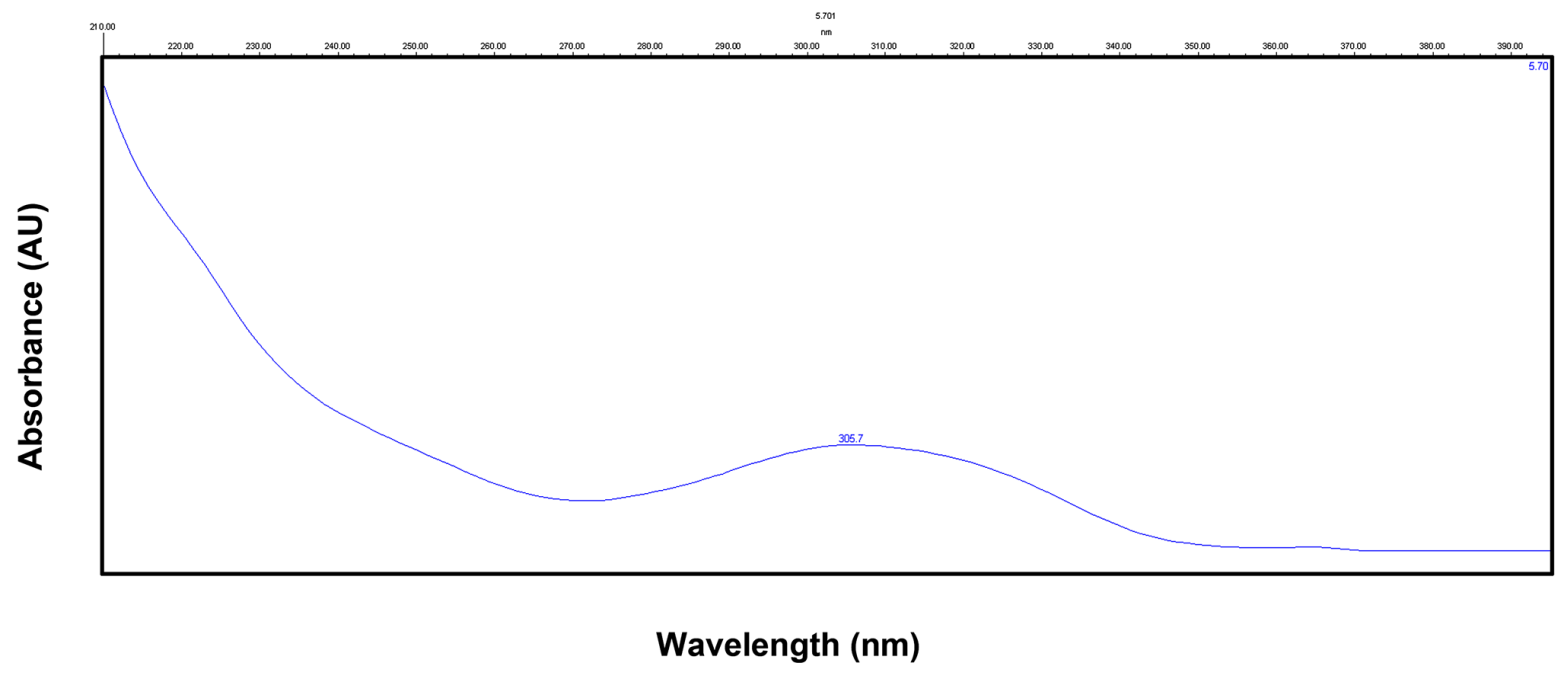
Absorbance spectrum of a standard solution of lamotrigine

**Fig. 3:**
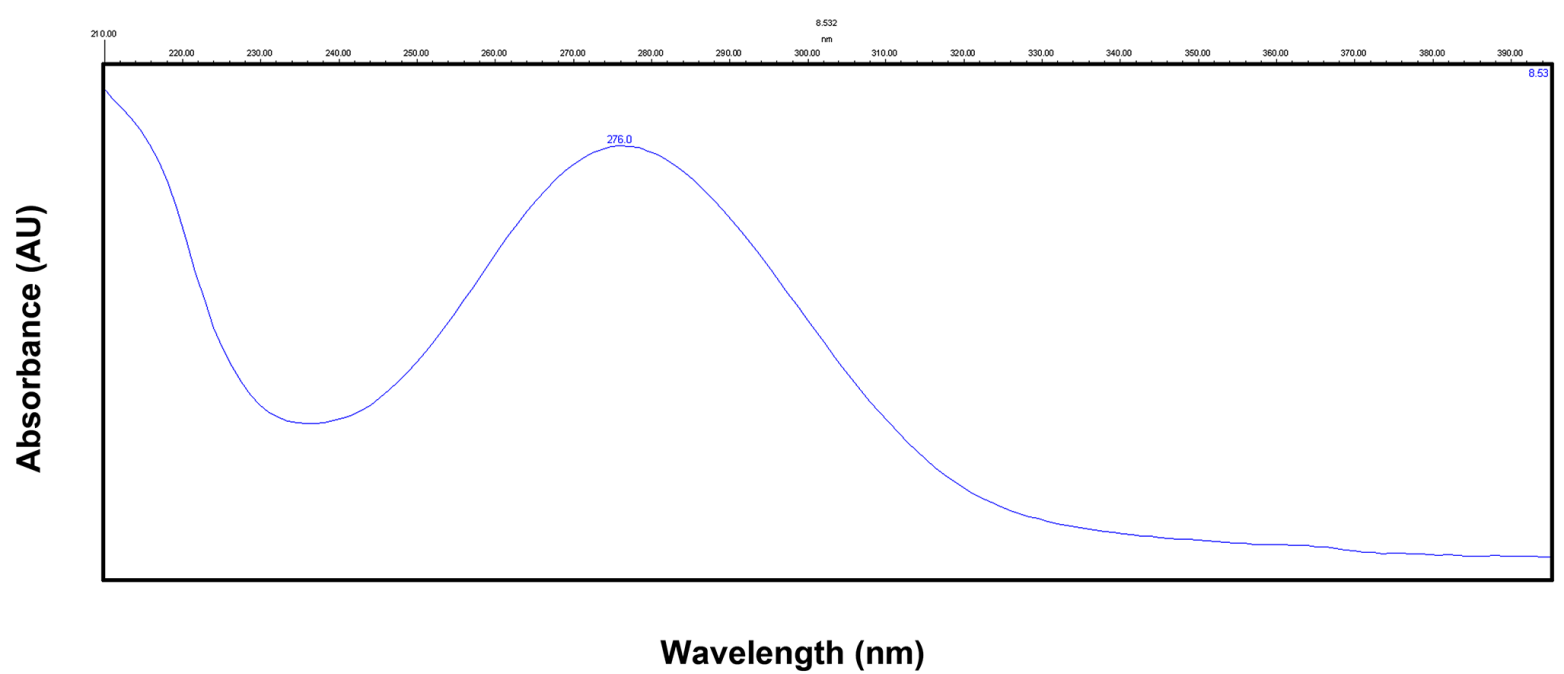
Absorbance spectrum of a standard solution of chloramphenicol

As mentioned before, an isocratic elution was applied at a flow rate of 1 ml/min with a mobile phase consisting of acetonitrile (30%) and 1 mM phosphate buffer (70%) (pH 6.5). The injection volume was 20 µl and run time was 10 min. Chromatographic separation was carried out on a reversed-phase column. These chromatographic conditions provided optimal separation of LTG and IS, with retention times (*t*_*R*_) of 5.75 min and 8.53 min, respectively (Fig. 1). Detection was monitored at λ 305.7 and 276.0 nm for LTG (Fig. 2) and chloramphenicol (Fig. 3), respectively.

### Method validation

#### Selectivity

**Fig. 4:**
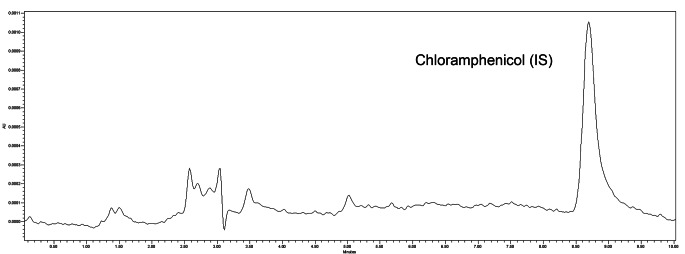
HPLC-PDA chromatogram of a blank human plasma sample spiked with the internal standard (chloramphenicol)

The analysis of ten blank plasma samples from different untreated subjects confirmed that there are no endogenous interferences affecting our analytical method. A chromatogram of a blank human plasma sample spiked with internal standard is shown in Fig. 4.

### Linearity

**Fig. 5:**
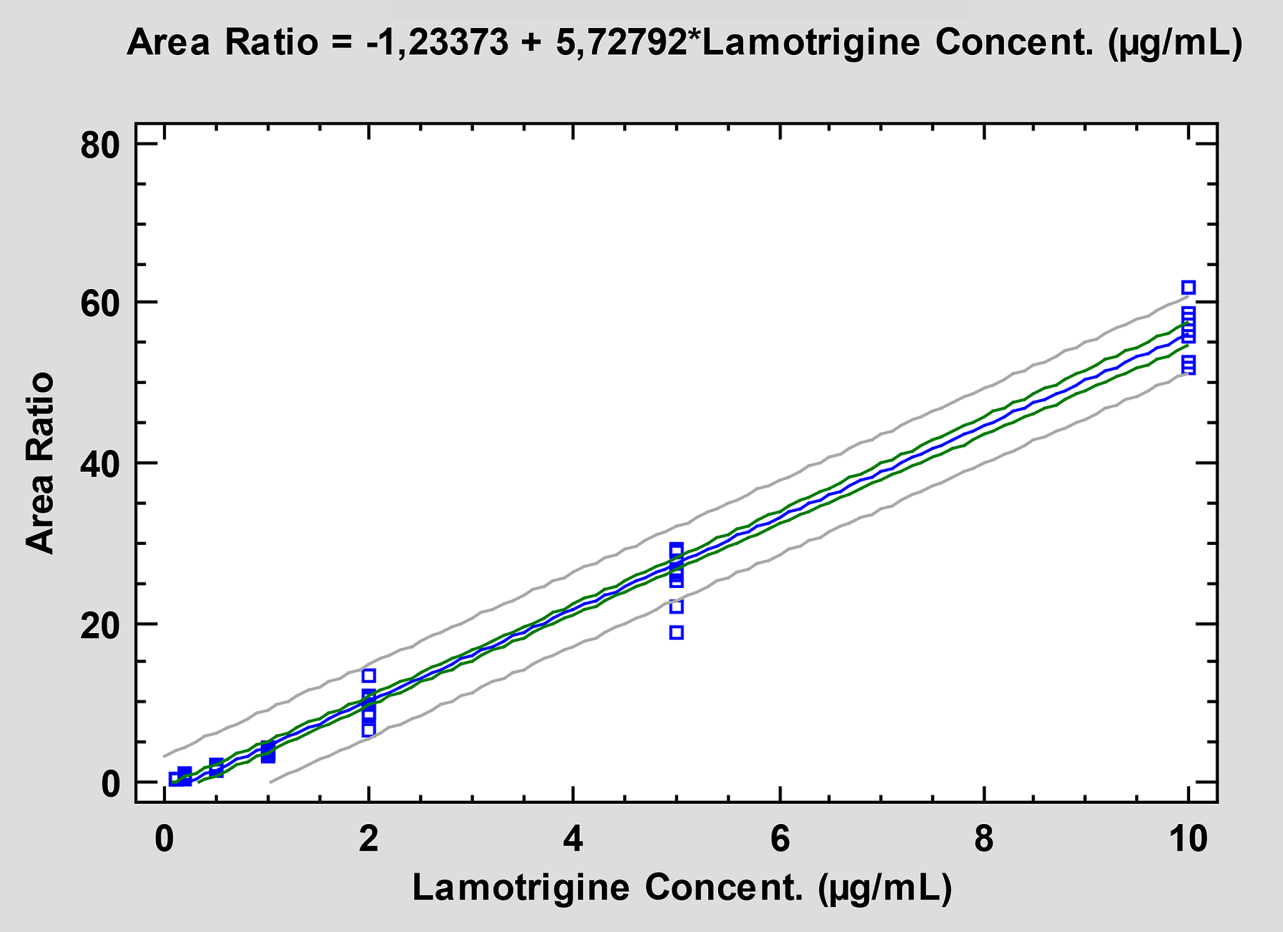
Calibration curve for LTG in human plasma

The calibration curves obtained for human plasma were linear within the concentration range selected (from 0.1 to 10 µg/ml) and showed a consistent correlation between the LTG/IS peak area ratios and the corresponding nominal concentration of LTG (*r* = 0.9934). The linear regression equation of the calibration curves for LTG in human plasma was y = -1.23373 + 5.72792x (Fig. 5).

### Limits of detection and quantification

The LOD, established as the lowest concentration giving a signal-to-noise ratio of 3, was 0.04 µg/ml. The LOQ, defined as the lowest concentration of LTG that can be measured with acceptable precision and accuracy, and calculated at a signal-to-noise ratio of 10, was found to be 0.1 µg/ml.

### Precision and accuracy

The intraday and interday precision and accuracy results obtained in human plasma at three concentration levels (QC_1_ = 0.1 µg/ml, QC_2_ = 1 µg/ml, and QC_3_ = 10 µg/ml), are shown in Table 1. The intra and interday precision values (%RSD) did not exceed 9.0%, and the intra and interday accuracy values (% *bias*) ranged from − 7.6 to 10.1%.

**Table 1:** Intra and interday precision (% RSD) and accuracy (% *bias*) values for LTG in human plasma at low (QC_1_), medium (QC_2_) and high (QC_3_) concentrations (*n* = 5)

C_nominal_^a^ (µg/ml)	Intraday			Interday		
	C_experimental_^b^ (mean ± SD^c^) (µg/ml)	Precision (% RSD^d^)	Accuracy (% *bias*)	C_experimental_ (mean ± SD) (µg/ml)	Precision (% RSD)	Accuracy (% *bias*)
QC_1_ 0.1	0.110 ± 0.001	1.3	10.1	0.102 ± 0.009	9.0	3.8
QC_2_ 1	0.989 ± 0.035	3.5	-1.2	0.924 ± 0.077	8.3	-7.6
QC_3_ 10	10.592 ± 0.285	2.7	5.9	10.141 ± 0.143	1.4	1.4

^a^Nominal concentration

^b^Experimental concentration

^c^Standard deviation

^d^Relative standard deviation

### Recovery

The LTG recovery results in human plasma were obtained by testing three different concentration levels (low QC_1_, medium QC_2_, and high QC_3_) (*n* = 5). The results, included in Table 2, show a recovery ≥ 98.9%.

**Table 2:** Recovery (values in percentage) of lamotrigine from human plasma at low (QC_1_), medium (QC_2_) and high (QC_3_) concentrations (*n* = 5)

	C_nominal_^a^ (µg/ml)	Recovery (%)	
		Mean ± SD	RSD (%)
QC_1_	0.1	110.0 ± 1.4	1.3
QC_2_	1.0	98.9 ± 3.5	3.5
QC_3_	10.0	105.9 ± 2.9	2.7

^a^Nominal concentration

### Method application

**Fig. 6:**
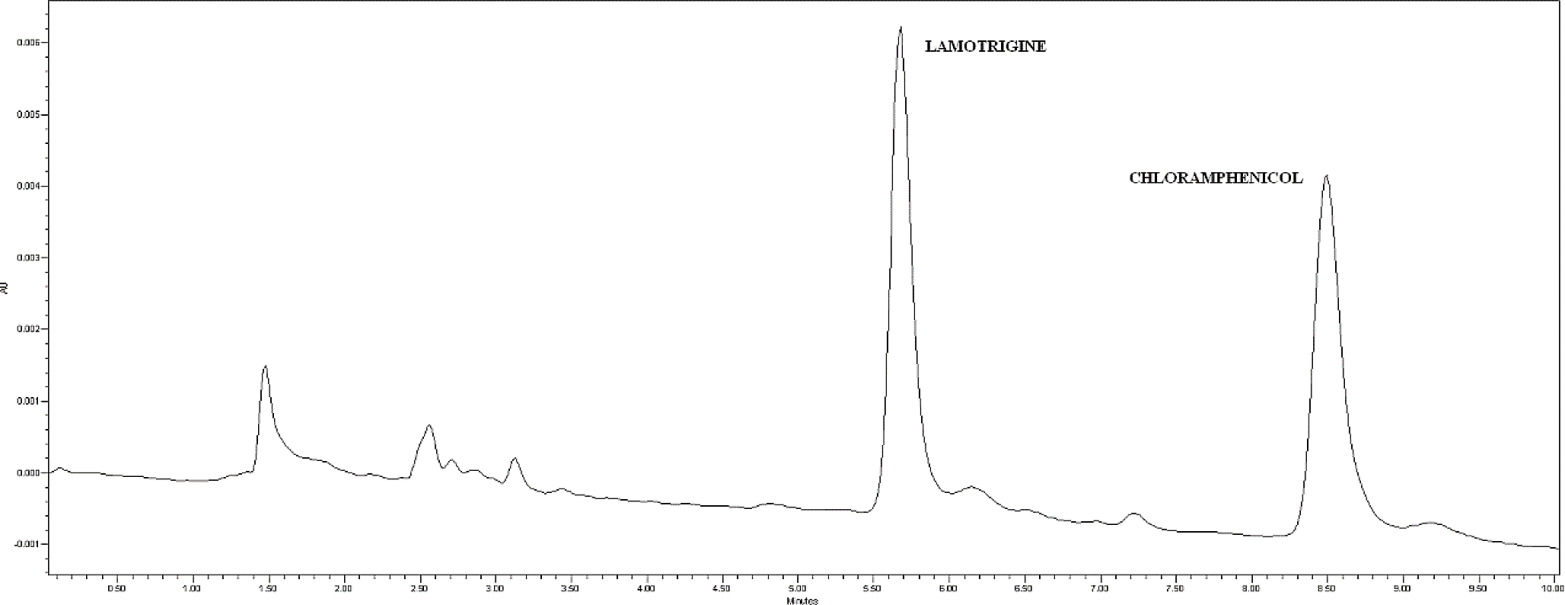
HPLC-PDA chromatogram of a postmortem sample received at the Forensic Toxicology Service of the Forensic Sciences Institute of Santiago de Compostela (Spain) and analyzed by the developed and validated method

The proposed method was applied to eleven human postmortem blood samples sent to our laboratory for analysis. Table 3 shows the results obtained in terms of LTG concentration and additional information such as sex, age, and the cause of death suspected by medical examiner based on the history and autopsy. Table 3 also lists other drugs identified and quantified in blood. Lamotrigine concentrations quantified ranged from 0.29 µg/ml (relatively low level) to 8.99 µg/ml (relatively high level). The chromatogram of one of these real postmortem samples is shown in Fig. 6.

**Table 3:** Results of analysis of postmortem blood samples from forensic cases and additional information

Case	Sex	Age (years)	Cause of death	LTG concentration (µg/ml)	Other compounds (concentration)
1	Female	27	Drug poisoning suicide	3.04	Venlafaxine (3.5 µg/ml) Sertraline (2.63 µg/ml) Paracetamol (65.6 µg/ml) Olanzapine (1.34 µg/ml)
2	Female	33	Drug poisoning suicide	4.59	Fluoxetine (1.08 µg/ml) Methadone (0.18 µg/ml)
3	Male	53	Drug Overdose	8.99	Pregabalin (25.5 µg/ml) Levomepromazine (0.25 µg/ml)
4	Female	34	Natural	1.26	─
5	Female	46	Drug poisoning suicide	7.36	Quetiapine (2.0 µg/ml) Lorazepam (0.24 µg/ml) Ethyl alcohol (0.46 g/l)
6	Male	79	Accidental fall	0.29	─
7	Female	56	Drug poisoning suicide	3.69	Sertraline (0.25 µg/ml) Carbamazepine (1.33 µg/ml)
8	Male	40	Natural	0.95	─
9	Male	63	Drug poisoning suicide	2.46	Gabapentin (7.16 µg/ml) Venlafaxine (0.15 µg/ml) Clomipramine (0.13 µg/ml) Diazepam (0.28 µg/ml) Oxazepam (0.08 µg/ml) Nordiazepam (0.42 µg/ml)
10	Male	47	Drug poisoning suicide	2.46	Carbamazepine (0.44 µg/ml) Oxazepam (0.5 µg/ml) Levetirazetam (14.7 µg/ml)
11	Male	50	Traffic accident	1.22	Quetiapine (0.23 µg/ml) Clomipramine (0.05 µg/ml)

## Discussion

The sample extraction procedure was optimized after a comparative study of different extraction procedures. Among those published, we tested the SPE procedure reported by Bugamelli et al. [27] and observed a better LTG peak area than those obtained by LLE and IL-DLLME procedures. In turn, the chloramphenicol peak area obtained by SPE procedure was very low. The IL-DLLME procedure [15] undetected chloramphenicol in our chromatographic conditions. Finally, LLE was selected and certain experimental variables such as pH, sample amount and extraction solvent were optimized. Our LLE procedure uses smaller sample and solvent volumes than other LLE procedures previously reported [10]. The proposed extraction procedure is simple, rapid, and requires small volumes not only of buffer (250 µl) but also of extraction solvent (2 ml of ethylacetate).

Different compounds have been used and reported as internal standards. The structures of some of them were similar to that of lamotrigine, whereas other internal standards showed not similar structures. Many compounds used as internal standard were not readily available. Some of the reported internal standards are drugs used in clinical practice, therefore, it is possible that these compounds or their metabolites are present in plasma samples before addition of any other internal standards. Some authors propose chloramphenicol, an antibiotic not currently prescribed in humans, as internal standard [11, 12, 14, 28]. Chloramphenicol was successfully tested and used as internal standard in our method.

Chromatographic separation was carried out successfully in 10 min with an injection volume of 20 µl. No endogenous interferences were observed when analyzing plasma samples. The calibration curves were linear within the concentration range selected, that is from 0.1 µg/ml to 10 µg/ml. The LOD was 0.04 µg/ml, lower than those previously reported by other authors [6, 7, 11, 12]. The LOQ was found to be 0.1 µg/ml. This LOQ was better than that reported in Refs [6, 7, 12]. or the same as that from Refs [16, 28]. achieved by other HPLC methods published in the literature. The intra and interday precision values and the intra and interday accuracy values fulfilled the acceptance criteria of FDA recommendations. The results showed an optimal extraction efficiency (recovery ≥ 98.9%).

The proposed method was successfully applied to eleven postmortem samples, sent to our laboratory for analysis. The application of the method to this group of real case samples shows its usefulness for analysis in any forensic toxicology laboratory. The interpretation of postmortem lamotrigine concentrations is not fully elucidated [29]. The blood lamotrigine concentration consistent with therapeutic use ranged from 0.9 µg/ml to 7.2 µg/ml, as it was reported in postmortem blood samples [29]. A range from 2 to 14 µg/ml has also been reported and applied as therapeutic one for the interpretation of results in real cases [30]. In our series of eleven cases involving lamotrigine, the cause of death in seven of them was attributed by the medical examiner to drug poisoning in combination with other substances. In these seven cases, blood concentrations of lamotrigine ranged from 2.46 µg/ml to 8.99 µg/ml. In the remaining four cases, the medical examiner proposed other causes of death which were; natural origin in two cases showing lamotrigine levels of 1.26 µg/ml and 0.95 µg/ml and no evidence of other associated medication, accidental fall in one case finding 0.29 µg/ml of lamotrigine and no other medication, and a traffic accident with a concentration of 1.22 µg/ml associated with quetiapine and clomipramine. Therefore, we found postmortem blood levels of lamotrigine ranging from 0.29 µg/ml to 1.26 µg/ml in cases whose cause of death was not attributed to drug poisoning.

## Conclusions

A simple HPLC-PDA method for the quantitative determination of lamotrigine in plasma was developed and validated. Different extraction procedures were tested and compared, and a liquid-liquid extraction procedure was finally selected. The proposed LL extraction procedure uses small amounts of buffer and solvent. The method validation, carried out according to the FDA guidelines, demonstrated good linearity, high sensitivity, precision, accuracy and recovery. The LOQ of this validated method was 0.1 µg/ml and the LOD was 0.04 µg/ml. The applicability of this method was verified using real postmortem blood samples. The proposed method is suitable for therapeutic drug monitoring and forensic toxicology analysis. In postmortem cases, the interpretation of the results remains to be fully elucidated. Mixed-drug toxicity and the time that may have elapsed between drug administration and the occurrence of death are factors, among others, to be taken into account.

### Key points

1. The proposed extraction method uses small volumes of buffer and ethylacetate.
2. Our HPLC method shows good linearity, sensitivity, precision, accuracy and recovery.
3. The developed and validated HPLC-PDA method is linear between 0.1 and 10 µg/ml.
4. The proposed method was successfully applied to forensic blood samples.
